# Focal Adhesion Genes Refine the Intermediate-Risk Cytogenetic Classification of Acute Myeloid Leukemia

**DOI:** 10.3390/cancers10110436

**Published:** 2018-11-13

**Authors:** Victor Pallarès, Montserrat Hoyos, M. Carmen Chillón, Eva Barragán, M. Isabel Prieto Conde, Marta Llop, Aïda Falgàs, María Virtudes Céspedes, Pau Montesinos, Josep F. Nomdedeu, Salut Brunet, Miguel Ángel Sanz, Marcos González-Díaz, Jorge Sierra, Ramon Mangues, Isolda Casanova

**Affiliations:** 1 Biomedical Research Institute Sant Pau (IIB Sant Pau), Hospital de la Santa Creu i Sant Pau, Sant Antoni Maria Claret 167, Pavelló 11, 2n pis, 08025 Barcelona, Spain; mpallaresl@santpau.cat (V.P.); mhoyos@santpau.cat (M.H.); AFalgas@santpau.cat (A.F.); mcespedes@santpau.cat (M.V.C.); icasanova@santpau.cat (I.C.); 2 Department of Hematology, Hospital de la Santa Creu i Sant Pau, Mas Casanovas nº 90, 08041 Barcelona, Spain; jnomdedeu@santpau.cat (J.F.N.); sbrunet@santpau.cat (S.B.); 3 Servicio de Hematología, IBSAL-Hospital Universitario, Centro de Investigación del Cáncer (CIC)-IBMCC, Centro de Investigación Biomédica en Red de Cáncer (CIBERONC), Universidad de Salamanca, 37007 Salamanca, Spain; chillon@usal.es (M.C.C.); i.prietoconde@usal.es (M.I.P.C.); margondi@usal.es (M.G.-D.); 4 Hematology Department, Hospital Universitari i Politècnic La Fe, Department of Medicine, University of Valencia, and Centro de Investigación Biomédica en Red de Cáncer, Instituto Carlos III, 46026 Valencia, Spain; barragan_eva@gva.es (E.B.); llop_mar@gva.es (M.L.); montesinos_pau@gva.es (P.M.); msanz@uv.es (M.Á.S.); 5 CIBER en Bioinginiería, Biomateriales y Nanomedicina (CIBER-BBN), 08025 Barcelona, Spain; 6 Josep Carreras Leukemia Research Institute, 08021 Barcelona, Spain; 7 Hematology Department, Universitat Autònoma de Barcelona, 08193 Barcelona, Spain

**Keywords:** acute myeloid leukemia, PTK2B, LYN, PTK2, prognostic factor, intermediate-risk

## Abstract

In recent years, several attempts have been made to identify novel prognostic markers in patients with intermediate-risk acute myeloid leukemia (IR-AML), to implement risk-adapted strategies. The non-receptor tyrosine kinases are proteins involved in regulation of cell growth, adhesion, migration and apoptosis. They associate with metastatic dissemination in solid tumors and poor prognosis. However, their role in haematological malignancies has been scarcely studied. We hypothesized that *PTK2/FAK*, *PTK2B/PYK2*, *LYN* or *SRC* could be new prognostic markers in IR-AML. We assessed *PTK2*, *PTK2B*, *LYN* and *SRC* gene expression in a cohort of 324 patients, adults up to the age of 70, classified in the IR-AML cytogenetic group. Univariate and multivariate analyses showed that *PTK2B*, *LYN* and *PTK2* gene expression are independent prognostic factors in IR-AML patients. *PTK2B* and *LYN* identify a patient subgroup with good prognosis within the cohort with non-favorable FLT3/NPM1 combined mutations. In contrast, *PTK2* identifies a patient subgroup with poor prognosis within the worst prognosis cohort who display non-favorable FLT3/NPM1 combined mutations and underexpression of *PTK2B* or *LYN*. The combined use of these markers can refine the highly heterogeneous intermediate-risk subgroup of AML patients, and allow the development of risk-adapted post-remission chemotherapy protocols to improve their response to treatment.

## 1. Introduction

Acute myeloid leukemia (AML) is a group of heterogeneous malignancies, all exhibiting the common feature of the infiltration of bone marrow, blood, and other tissues by proliferative immature cells of the hematopoietic system [1,2,3].

The AML classification has evolved over the last decades; currently patients are classified into favorable, intermediate, or adverse risk groups, according to cytogenetic and molecular features to adapt their therapy after remission [2]. Favorable and adverse patient groups are well characterized by specific chromosomal alterations, and usually receive well-established treatment protocols. However, the intermediate-risk cytogenetics group (IR-AML) patients are very heterogeneous, present a widely diverse outcome, and therapeutic approaches are diverse, since they are still not well established [4]. This uncertainty concerning therapy impairs survival of many IR-AML patients. Hence, there is a need for new prognostic factors capable of stratifying this patient subgroup to improve their outcome. To that purpose, over the last years, there has been an intense search for novel genetic alterations in an attempt to improve IR-AML patient stratification. However, only the molecular markers NPM1, CEBPA and FLT3 have been incorporated into clinical practice [2,5,6]. Thus, a combination of mutated NPM1 and non-mutated FLT3/Internal tandem duplication (FLT3/ITD) associates with favorable outcome in IR-AML patients [7,8,9,10]. Likewise, biallelic CEBPA mutations predict a relatively favorable outcome in patients without FLT3/ITD [11]. More recently, mutations of RUNX1, ASXL1 and TP53 have been associated with adverse outcome and are also included in the standard diagnostic work up [4]. Additionally, the identification of molecular alterations, such as IDH1, IDH2, TET2, DNMT3A, MLL-PTD or NRAS, allow a better stratification of a subset of IR-AML patients [12,13]. However, these factors have not been yet introduced in clinical practice. Thus, stronger prognostic indicators with higher stratification capacity regarding differences in clinical outcome remain to be identified.

The non-receptor tyrosine kinases (NRTKs) are cytoplasmic proteins that play important roles in migration, metabolism, adhesion, proliferation and cell differentiation [14]. Integrin engagement through the receptor tyrosine kinases (RTKs) triggers downstream signaling and activate these cell functions. Protein tyrosine kinases, such as focal adhesion kinase (FAK, also named PTK2) or proline-rich tyrosine kinase 2 (PYK2/PTK2B/RAFTK), and SRC family proteins, among others, participate in this complex signaling network. In the cytoplasm, the structurally similar FAK or PYK2 are phosphorylated in response to extracellular stimuli, such as hormones, growth factors, integrins or cytokines. On the other hand, SRC family members, such as SRC or LYN, have the capacity to stabilize focal adhesion complexes through the phosphorylation of FAK kinases [14,15,16]. Whereas FAK and SRC are expressed in a wide variety of tissues and cells, PYK2 and LYN are predominantly expressed in hematopoietic cells [17,18]. In hematologic malignancies, the role of these protein kinases has been poorly studied. In addition, whereas FAK was found associated with poor outcome in AML [19,20,21], no studies have addressed SRC, PYK2 or LYN expression as possible prognostic factors in AML, and particularly in IR-AML patients.

We here studied whether focal adhesion genes, such as *PTK2* (gene encoding for the FAK protein), *PTK2B* (gene encoding for the PYK2 protein), *SRC* or *LYN* could have prognostic impact and correlate with survival in IR-AML patients. We demonstrate, for the first time, that the expression of *PTK2B* and *LYN* identify a patient subgroup within AML intermediate-risk patients with a better prognosis, whereas *PTK2* expression (together with *PTK2B* or *LYN* underexpression) identifies patients with a poorer prognosis.

## 2. Results

### 2.1. Patient Characteristics

**Figure 1:**
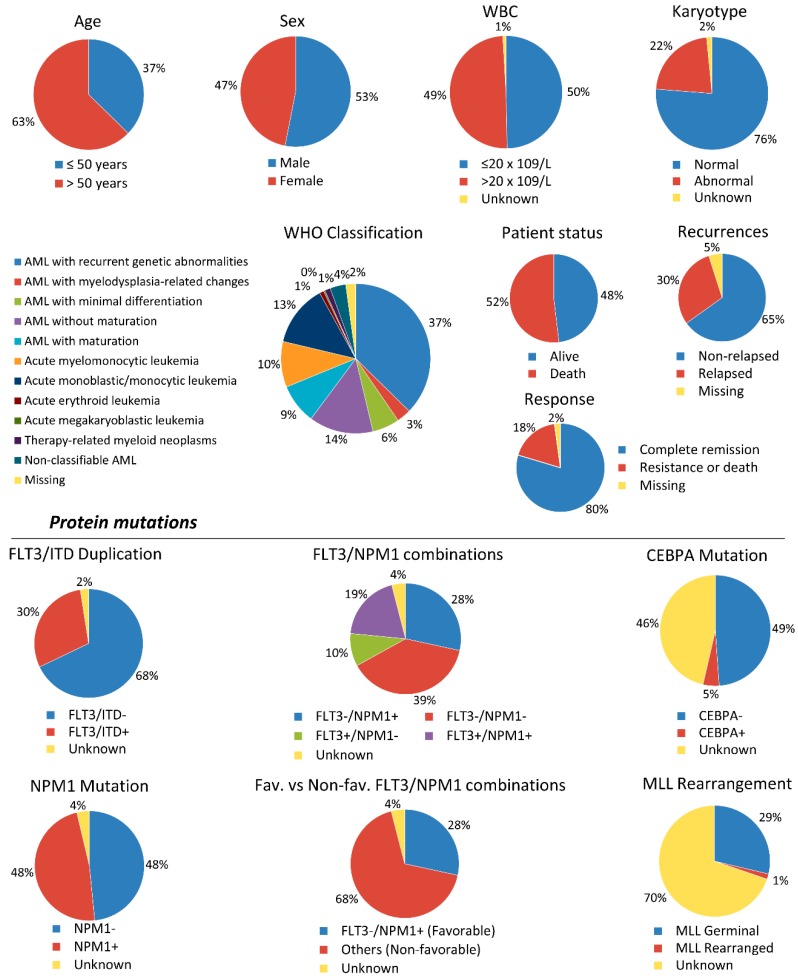
Main clinical characteristics of the studied cytogenetic intermediate-risk acute myeloid leukemia (IR-AML) patient cohort. Results are presented in pie graphs showing percentage of patients for each feature or parameter. WBC; white blood cells. FAB; French-American-British. WHO; World Health Organization. Fav; favorable. Non-fav; non-favorable.

The main characteristics of patients with IR-AML (N = 324) are shown in Figure 1 (Table S1). Median age was 55 with a 37% of the patients being 50 years old or younger. Fifty three percent were men, and 76% of patients presented normal karyotype. At the end of the study, 48% of patients (156/324) were alive, 30% (97/324) had relapsed, and 52% (168/324) were dead. 

Alive patients had a median follow-up of 55 months (Table S2), and relapsed patients after complete remission (CR) had a median follow-up of 11 months, with a range between 0.4 and 68 months. At five years, overall survival (OS), disease-free survival (DFS) and cumulative incidence of relapse (CIR) were 45.4 ± 2.9%, 44.9 ± 3.2% and 36.7 ± 3.1%, respectively (Table S2). Finally, the analysis of clinical variables and molecular alterations using time-dependent outcome endpoints showed that patients older than 50 had worst OS and DFS than younger patients (Table S2). Moreover, patients with favorable FTL3/NPM1 combination (patients that presented FLT3 wild type (wt) and NPM1 mutated; FLT3^−^/NPM1^+^) showed better OS and DFS, and lower CIR than patients with non-favorable FLT3/NPM1 combinations (patients with FLT3/ITD mutations and NPM1 mutated or wt; FLT3^+^/NPM1^+^ and FLT3^+^/NPM1^−^; and those who presented FLT3 and NPM1 wt; FLT3^−^/NPM1^−^) (Table S2).

### 2.2. PTK2B and LYN Overexpression Are Independent Favorable Prognostic Factors for OS and DFS, Whereas Overexpression of PTK2B Prognosticates also a Favorable CIR in IR-AML Patients

Dichotomized clinical variables, such as age, sex, white blood cells (WBC) and molecular alterations, including FLT3/ITD duplication, NPM1 mutation, FLT3/NPM1 combined mutations (favorable vs. non-favorable) and karyotype, as well as *PTK2B*, *LYN*, *PTK2* and *SRC* expression were assessed in an univariate analysis using a Cox regression test individually for each variable. Following, a multivariate analysis was performed, including only the variables reaching *p*-value lower than 0.100 in the univariate analysis. These variables were introduced in a Cox regression test simultaneously to assess its independence as prognostic factors.

In the univariate analysis (Table 1), Cox regression and Fine and Gray’s tests showed that age over 50 and non-favorable FLT3/NPM1 combinations (FLT3^+^/NPM1^−^, FLT3^−^/NPM1^−^ and FLT3^+^/NPM1^+^) were associated with lower OS (*p* < 0.001, HR = 1.947; *p* = 0.014, HR = 1.586, resp.) and lower DFS (*p* = 0.005, HR = 1.639; *p* = 0.014, HR = 1.617, resp.). In contrast, *PTK2B* and *LYN* overexpression were associated with higher OS (*p* = 0.008, HR = 0.589; *p* = 0.014, HR = 0.621, resp.) and higher DFS (*p* = 0.005, HR = 0.552; *p* = 0.007, HR = 0.569, resp.).

Regarding CIR (Table 2), non-favorable FLT3/NPM combinations or *PTK2B* overexpression were associated with higher and lower CIR, respectively (*p* = 0.007, HR = 1.920; *p* = 0.002, HR = 0.426, resp.). *SRC* and *PTK2* expression showed no association with OS, DFS or CIR in these univariate analyses.

All variables showing statistical significance in the univariate analyses maintained their significance in the multivariate analyses for OS, DFS and CIR (Table 1 and Table 2). 

### 2.3. Kaplan-Meier Curves Confirm the Favorable Prognosis of PKT2B or LYN Overexpression Regarding IR-AML Patient OS, DFS or CIR

**Figure 2:**
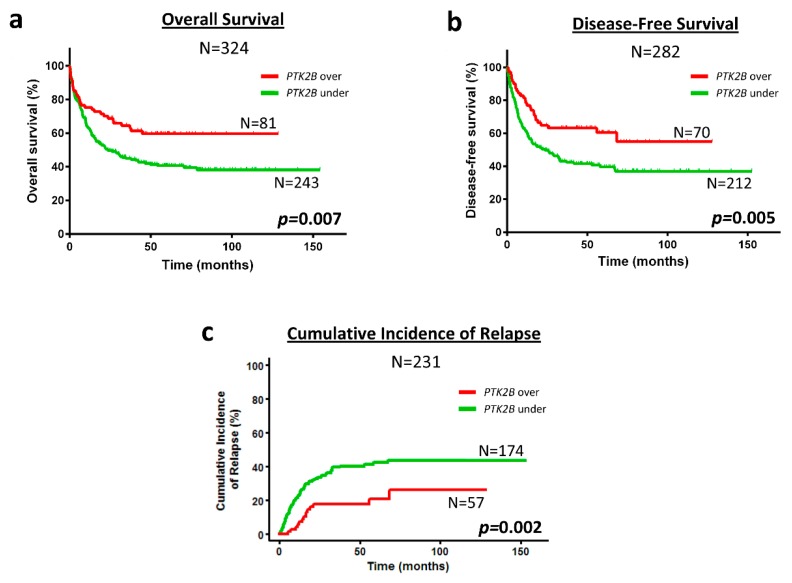
*PTK2B* overexpression associates with favorable prognosis regarding OS, DFS and CIR in cytogenetic IR-AML patients. Kaplan-Meier curves represent OS (**a**) DFS (**b**) and CIR (**c**), depending on the *PTK2B* expression. Black line and gray line indicate *PTK2B* overexpressed and underexpressed, respectively. Log-rank test for OS and DFS, and Gray test for CIR were used to analyze the statistical significance. *p* < 0.05 was considered statistically significant (bold values). Over; overexpression. Under; underexpression.

**Figure 3:**
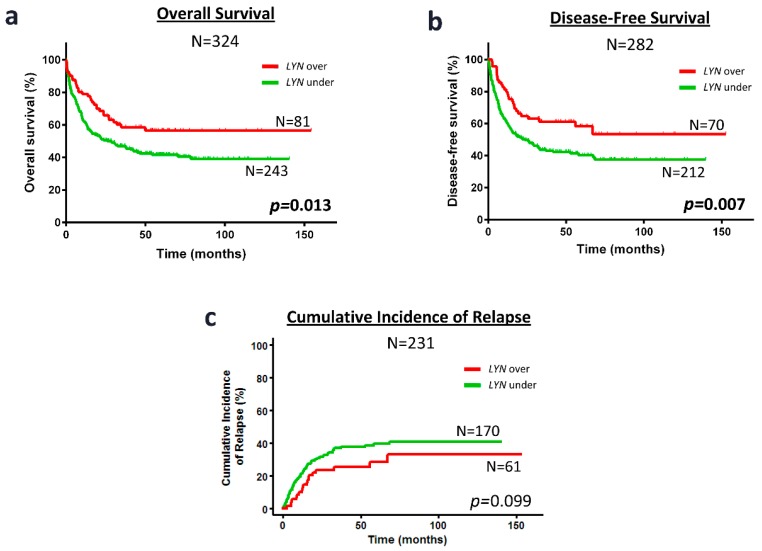
*LYN* overexpression associates with favorable prognosis regarding OS and DFS in cytogenetic IR-AML patients. Kaplan-Meier curves represent OS (**a**) DFS (**b**) and CIR (**c**), depending on the *LYN* expression. Black line and gray line indicate *LYN* overexpressed and underexpressed, respectively. Log-rank test for OS and DFS, and Gray test for CIR were used to analyze the statistical significance. *p* < 0.05 was considered statistically significant (bold values).

Kaplan-Meier analysis showed that patients with overexpression of *PTK2B* presented better prognosis than patients with low *PTK2B* expression regarding OS (59.7 ± 5.7 vs. 38.1 ± 3.6%, *p* = 0.007), DFS (54.9 ± 7.7 vs. 37.0 ± 3.9%, *p* = 0.005) and CIR (26.2 ± 7.4 vs. 43.6 ± 3.9%, *p* = 0.002) (Figure 2). Similarly, patients with overexpression of *LYN* had better prognosis than patients with *LYN* underexpression in OS (56.6 ± 5.8 vs. 39.1 ± 3.6%, *p* = 0.013) and DFS (53.5 ± 7.5 vs. 37.6 ± 3.9%, *p* = 0.007) (Figure 3a,b). However, the differences in CIR between patients that overexpressed or underexpressed *LYN* were not statistically significant (33.3 ± 7.4 vs. 41.0 ± 3.8%, *p* = 0.099) (Figure 3c). No differences were found in the Kaplan-Meier analyses between patients with over or underexpression of *SRC* or *PTK2*, and survival.

### 2.4. Overexpression of PTK2B or LYN Associated with Higher Rate of Alive Patients and Lower Rate of Relapse in Cytogenetic IR-AML Patients

The possible association between *PTK2B* and *LYN* and several variables, such as age, sex, WBC, karyotype, FLT3/ITD duplication, NPM1 mutation, FLT3/NPM1 combined mutations, recurrences (relapsed or no relapsed) and patient status, at the end of follow up (alive or death), was evaluated in this cohort. Most analyzed variables showed no association with *PTK2B* or *LYN* expression (Table 3).

Only patient status and recurrence showed a significant association with *PTK2B* or *LYN* expression level. Thus, the percent of alive patients at the end of the study was higher among those overexpressing *PTK2B* (*p* = 0.007) or *LYN* (*p* = 0.029). Moreover, the percent of patients on relapse was lower among those overexpressing *PTK2B* (*p* = 0.007).

In addition, we performed a correlation analysis between *PTK2B* and *LYN* expression. We found that the expression of these two genes correlated positively in the patient sample (*ρ* = 0.672; *p* < 0.001). 

### 2.5. Overexpression of PTK2B or LYN in Cytogenetic IR-AML Patients with Non-Favorable FLT3/NPM1 Combinations Is as Good in Prognosticating Survival or Recurrence as the Favorable FLT3/NPM1 Combination 

Once we found *PTK2B* or *LYN* overexpression having independent prognostic capacity in cytogenetic IR-AML patients (N = 324), we explored their prognostic capacity in the patient subgroup having non-favorable FLT3/NMP1 combinations (N = 219). 

The new guide from the European LeukemiaNet (ELN) for AML risk stratification and recent studies [4,7,8,9,10] have established that patients with a combination of mutated NPM1 without FLT3/ITD had to be considered of favorable risk. On this basis, we performed analyses focusing on validating this association in our cohort (N = 324). Both Kaplan-Meier and univariate/multivariate analyses showed that favorable FLT3/NPM1 combination (FLT3^−^/NPM1^+^) had better prognosis than the rest of FLT3/NPM1 combinations (FLT3^+^/NPM1^−^, FLT3^−^/NPM1^−^ and FLT3^+^/NPM1^+^) regarding OS, DFS and CIR (Table 1, Table 2 and Table S2); therefore, being FLT3^−^/NPM1^+^ identified as an independent favorable prognostic factor in our IR-AML cohort.

**Figure 4:**
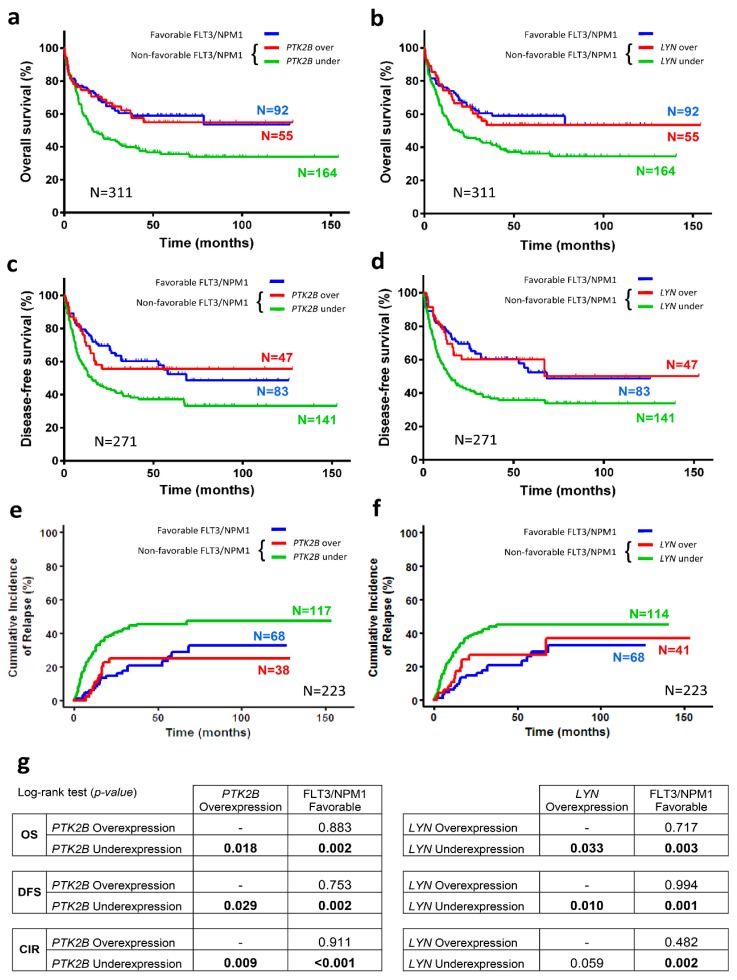
PTK2B or LYN overexpression identifies a subgroup of patients with cytogenetic IR-AML and non-favorable FLT3/NPM1 combinations that show favorable OS, DFS and CIR. Kaplan-Meier curves represent survival depending on the PTK2B (**a**, OS; **c**, DFS; **e**, CIR) or LYN (**b**, OS; **d**, DFS; **f**, CIR) expression. Red line and green line indicate PTK2B or LYN overexpressed and underexpressed, respectively. Blue line indicates favorable FLT3/NPM1 combination prognosis. Log-rank test for OS and DFS, and Gray test for CIR were used to analyze the statistical significance and are represented in the table (**g**). *p* < 0.05 was considered statistically significant (bold values).

Next, we focused our analyses in the IR-AML patient subgroup with non-favorable FLT3/NPM combinations (N = 219) who present heterogeneous clinical outcomes. Kaplan-Meier analysis showed that patients in this subgroup who overexpressed *PTK2B* presented better prognosis than patients with low expression of *PTK2B* in OS (55.0 ± 7.1 vs. 34.0 ± 4.1%, *p* = 0.018), DFS (55.7 ± 7.4 vs. 33.3 ± 4.7%, *p* = 0.029) and CIR (25.0 ± 6.7 vs. 47.6 ± 4.7%, *p* = 0.009) (Figure 4a,c,e,g). In the same vein, patients with overexpression of *LYN* had better prognosis than patients with underexpression of *LYN* in OS (53.5 ± 7.1 vs. 34.6 ± 4.1%, *p* = 0.033) and DFS (50.1 ± 11.0 vs. 33.9 ± 4.4%, *p* = 0.010) (Figure 4b,d,g). Moreover, despite differences between patients who overexpressed and who underexpressed *LYN* were not statistically significant regarding CIR, they showed a trend toward significance (36.9 ± 11.6 vs. 44.9 ± 4.4%, *p* = 0.059) (Figure 4f,g). In addition, patients with overexpression of *PTK2B* or *LYN* showed no statistical differences in prognosticating OS, DFS or CIR, as compared with patients with favorable FLT3/NPM1 combination (N = 92) (Figure 4a–g). No differences were found in Kaplan-Meier analyses of *SRC* or *PTK2* expression. On the other hand, after log-rank test analyses of relevant clinical variables, only patients older than 50 had lower OS and DFS than younger patients in this patient subgroup (Table S3).

### 2.6. PTK2B or LYN Overexpression Are Independent Favorable Prognostic Factors for OS, DFS and CIR in Cytogenetic IR-AML Patients with Non-Favorable FLT3/NPM1 Combinations

To confirm the results obtained in Kaplan-Meier curves, we also performed univariate and multivariate Cox regression analyses to identify independent factors predictive of outcome, including as independent variables the expression of the focal adhesion genes in our cohort of IR-AML patients with non-favorable FLT3/NPM1 combinations (N = 219). 

Clinical variables, such as age, sex and WBC, and molecular variables. including FLT3/ITD duplication, NPM1 mutation, karyotype, as well as *PTK2B*, *LYN*, *PTK2* and *SRC* expression were assessed individually in the univariate analysis (a Cox regression test for each variable). Only variables with a *p*-value lower than 0.100 in the univariate analysis were included in the multivariate analysis (including clinical variables and gene expression at once in a Cox regression test).

In the univariate analysis, Cox regression and Fine and Gray’s tests showed that patients with age over 50 had lower OS and DFS (*p* = 0.001, HR = 1.913; *p* = 0.008, HR = 1.707, resp.) than younger patients (Table 4).

On the contrary, patients with overexpression of *PTK2B* or *LYN* presented better OS (*p* = 0.020, HR = 0.605; *p* = 0.035, HR = 0.625, resp.) and better DFS (*p* = 0.031, HR = 0.584; *p* = 0.012, HR = 0.527, resp.) than patients with these genes underexpressed (Table 4). Concerning CIR, only patients with overexpression of *PTK2B* were associated with better prognosis (*p* = 0.009, HR = 0.440), whereas *LYN* expression only showed a trend toward significance (Table 5). *SRC* or *PTK2* expression showed no association with survival or relapse in the univariate analyses (Table 4 and Table 5).

In the multivariate analyses (Table 4 and Table 5), *PTK2B* and *LYN* variables maintained the significant differences between groups, associating gene overexpression with better OS, DFS or lower CIR. 

In all the cases with statistical significance, age over 50 had a HR above 1, whereas the HR for *PTK2B* or *LYN* overexpression was lower than 1. 

### 2.7. In Cytogenetic IR-AML Patients with Non-Favorable FLT3/NPM1 Combinations and PTK2B or LYN Underexpression, PTK2 Overexpression Identify a Subgroup with Unfavorable OS and DFS

Based on previous evidences establishing the FAK protein as a poor prognostic factor in AML patients [19,20,22], and the fact that *PTK2* (gene encoding for FAK protein) was not identified as prognostic factor for clinical outcome in IR-AML in our study, we decided to assess *PTK2* expression in the patient subset with worst prognosis in our series, which were those showing underexpression of *PTK2B* or *LYN* and non-favorable FLT3/NPM1 combinations (N = 164).

**Figure 5:**
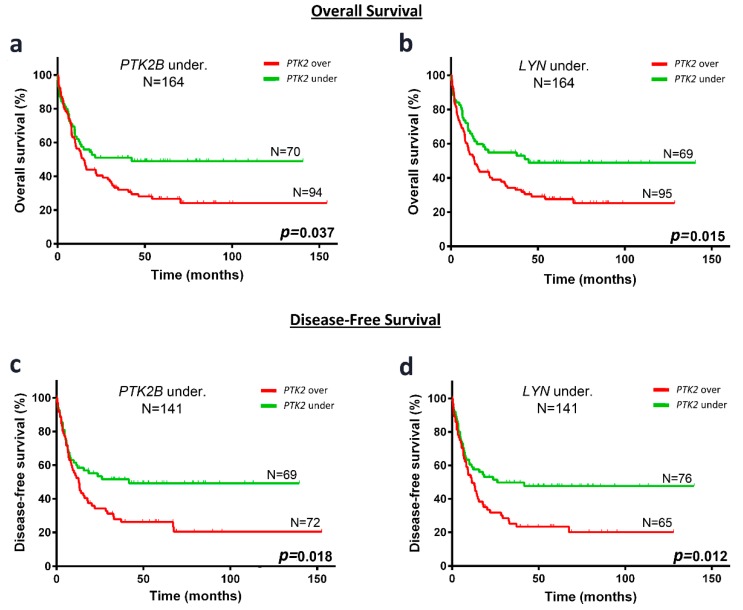
PTK2 overexpression associates with poor OS or DFS in cytogenetic IR-AML patients with non-favorable FLT3/NPM1 combinations that underexpress PTK2B or LYN. Kaplan-Meier curves represent survival depending on the PTK2 expression in PTK2B (**a**; OS, **c**; DFS) or LYN (**b**; OS, **d**; DFS) underexpressed patients. Black line and gray line indicate PTK2 overexpressed and underexpressed, respectively. Log-rank test for OS and DFS were used to analyze the statistical significance. *p* < 0.05 was considered statistically significant (bold values).

In the subgroup of patients with underexpressed *PTK2B* (Figure 5a,c), Kaplan-Meier survival curves showed that patients with *PTK2* overexpression had worst prognosis than those with low expression of *PTK2*, regarding OS (24.2 ± 5.0 vs. 49.0 ± 6.2%, *p* = 0.037) and DFS (20.4 ± 5.6 vs. 49.2 ± 6.4%, *p* = 0.018). Similar results were found in patients with underexpression of *LYN*, where patients overexpressing *PTK2* had worst prognosis than those with *PTK2* underexpression, regarding OS (25.3 ± 5.0 vs. 48.9 ± 6.4%, *p* = 0.015) and DFS (20.1 ± 5.6 vs. 47.6 ± 6.1%, *p* = 0.012) (Figure 5b,d).

### 2.8. A Multivariate Analysis Confirms PTK2 Overexpression as an Independent Prognostic Factor for OS and DFS in Cytogenetic IR-AML Patients with Non-Favorable FLT3/NPM1 Combinations and PTK2B or LYN Underexpression

Clinical variables, such as age, sex and WBC and molecular variables, including FLT3/ITD duplication, NPM1 mutation, karyotype, as well as *PTK2* expression were assessed in the univariate analysis (a Cox regression test individually for each variable). Only variables with a *p*-value lower than 0.100 were included in the multivariate analysis (including clinical variables and *PTK2* expression at once in a Cox regression test).

In patients with underexpression of *PTK2B* (N = 164) (Table 6), univariate analysis showed that patients older than 50 had worst OS and DFS (*p* = 0.010, HR = 1.717; *p* = 0.030, HR = 1.627, resp.) than younger patients. In addition, patients with *PTK2* overexpression had lower OS and DFS (*p* = 0.039, HR = 1.548; *p* = 0.019, HR = 1.686, resp.) than those with underexpression of *PTK2*. 

In the cohort of underexpressed *LYN* patients (N = 164) (Table 7), the Cox regression test showed that patients with overexpression of *PTK2* or older than 50 had worst prognosis regarding OS (*p* = 0.002, HR = 1.928; *p* = 0.016, HR = 1.669, resp.) and DFS (*p* = 0.015, HR = 1.722; *p* = 0.013, HR = 1.722, resp.) than those with underexpression of *PTK2* or younger, respectively. None of the assessed variables showed a significant association with CIR in the univariate analyses.

In the multivariate analyses (Table 6 and Table 7), the variable *PTK2* maintained its association with OS and DFS, whereas age only maintained its association with OS. As in the univariate analyses, patients older than 50 years had poor OS, whereas patients with *PTK2* overexpression had lower OS and DFS.

## 3. Discussion

In the last decades, novel molecular markers have improved AML patient stratification in order to adapt therapy to their prognosis. Although there are promising studies that stratified the IR-AML subgroup based on their molecular alterations [12], to reduce the number of patients included in this subgroup, more than a third of AML patients still show unpredictable outcomes. At present, only the analyses of NPM1, CEBPA and FLT3/ITD mutations have effectively been incorporated into routine prognostic stratification [6]. More recently, RUNX1, TP53 and ASXL1 mutations have been also associated with unfavorable outcome [4]. However, further studies must be carried out to better stratify the heterogeneous IR-AML patient group. We focused our study in the prognostic capacity of focal adhesion genes *PTK2B* (gene encoding for PYK2 protein), *LYN*, *SRC* and *PTK2* (gene encoding for FAK protein) in cytogenetic IR-AML patients.

### 3.1. PTK2B and LYN Overexpression Are Independent Prognostic Factors for Favorable Outcome in Cytogenetic IR-AML Patients

We identified *PTK2B* or *LYN* overexpression as independent favorable prognostic factors for OS, DFS and/or CIR in cytogenetic IR-AML patients, a relationship that was confirmed by Kaplan-Meier analysis by finding a higher rate of alive patients and lower rate of relapse in this patient subgroup. The capacity of *PTK2B* (gene encoding for PYK2 protein) or *LYN* overexpression to predict a favorable prognosis in our cohort could relay in the involvement of these proteins, PYK2 and LYN, in pro-apoptotic and differentiation processes; so that they identify a subset of patients with a more differentiated and more therapy responsive phenotype. 

The PYK2 protein is a member of the focal adhesion kinase (FAK) family that is predominantly expressed in neuronal and hematopoietic cells and regulates numerous and diverse cellular processes [23]. In agreement with our results, in normal monocytes, PYK2 expression correlates with differentiation and promotes apoptosis thereby controlling monocyte turnover [24]. Moreover, PYK2 expression has been also described in a majority of AML patients and its activation, by phosphorylation on Tyr-881 correlates with FAK expression. Interestingly, the functions of FAK and PYK2 in tumor cells are not necessarily redundant as in some settings they can play different or even opposing roles [25]. Also, consistently with our findings, PYK2 mediates apoptosis induced by alkylating agents or cytokines in normal monocytes and hematological malignant cells [26,27,28]. Moreover, PYK2 activation in leukemic cells induces growth arrest, substrate attachment and monocyte-like maturation when induced by phorbol esters [29]. In addition, dimethyl sulfoxide-induced PYK2 overexpression leads to the functional activation of granulocytes [30].

Among SRC family kinases, LYN is consistently expressed at a high level and constitutively activated in leukemic cells and also in the subcompartment enriched for leukemic stem cells. In normal cells, LYN is located in lipid rafts, where it is regulated by Cbp-CSK complex, but in leukemic cells it is distributed over the plasma membrane and also in the cytoplasm escaping from the Cbp-CSK negative regulation [31].

Regarding normal myeloid hematopoiesis, LYN is in general considered as a negative regulator, except for terminal maturation of erythroblasts [32]. Interestingly, in a LYN^−/−^ mouse model, loss of LYN function induces an increase of myeloid progenitors leading to tumor development. By contrast, mice bearing constitutively active LYN mutations did not exhibit leukemia development [33].

Similarly to PYK2, LYN overexpression associates with the induction of differentiation in cultured primary AML blasts [34]. Also, in normal myeloid differentiation, LYN activation associates with differentiation from hematopoietic stem cells or immature precursors into eosinophils [35] or erythrocytes [36], and is involved in signal transduction of mature normal hematopoietic cells, such as erythrocytes, platelets, mast cells and macrophages [16,31,37]. Moreover, in agreement with our results, LYN overexpression in primary AML cells associates with AML patients with favorable cytogenetic-risk [34].

### 3.2. PTK2B or LYN Overexpression Add Prognostic Value to the Cytogenetic IR-AML Patient Subgroup with Non-Favorable FLT3/NPM1 Combinations

Once we found that *PTK2B* or *LYN* overexpression had prognostic value in cytogenetic IR-AML patients, we further explored their ability to predict outcome in the patient subgroup with non-favorable FLT3/NMP1 combinations. We identified *PTK2B* or *LYN* overexpression as independent favorable prognostic factors for OS, DFS and CIR in the cytogenetic IR-AML patients bearing the different non-favorable FLT3/NPM1 combinations (FLT3^+^/NPM1^−^, FLT3^−^/NPM1^−^, FLT3^+^/NPM1^+^). Remarkably, the different non-favorable FLT3/NPM1 combinations showed no significant differences among them regarding outcome in our IR-AML cohort, confirming their heterogeneous clinical evolution. On this basis, we suggest that measuring *PTK2B* or *LYN* expression, in addition to the FLT3/NPM1 combinations, can improve patient stratification. We believe this is an important finding, since it identifies a subgroup of patients with favorable prognosis (as assessed by *PTK2B* or *LYN* expression) that would have been identified as of non-favorable outcome if using only FLT3/NPM1 criteria. 

### 3.3. Impact of the PTK2 Overexpression on the Prognosis of Cytogenetic IR-AML Patients with Non-Favorable FLT3/NPM1 Combinations that Underexpress PTK2B or LYN 

*PTK2* did not help in stratifying cytogenetic IR-AML when pooling together favorable and non-favorable FLT3/NMP1 combinations; however, it was useful when limiting the study to non-favorable FLT3/NMP1 combinations. Specifically, we found *PTK2* overexpression to be an independent prognostic factor that distinguishes a patient subgroup with poor outcome among those with IR-AML cytogenetics and non-favorable FLT3/NPM1 combinations, only when they also underexpress *PTK2B* or *LYN*. We believe this finding is important because it may identify a subgroup with very poor prognosis (patients with *PTK2* overexpression and *PTK2B* or *LYN* underexpression) within the highly heterogeneous patients bearing non-favorable FLT3/NPM1 combinations. 

This finding is consistent with FAK protein (encoded by the *PTK2* gene) overexpression being associated with poor prognosis in an AML patient cohort that included all risk subgroups [19]. The basis of their poor prognosis may relay in the aberrant FAK expression observed in primitive AML or leukemic stem cells (LSCs; CD45^lo^CD34^hi^CD38^−/lo^CD123^+^), which confers more migration capacity, while promoting their survival and enhancing their resistance to drugs, through FAK-mediated interaction with stroma [19,20,21,22]. Despite FAK is expressed in a wide variety of tissues and cells [17,18], it is not expressed in normal CD34+ hematopoietic precursor cells (HPCs); however, it is aberrantly expressed in primitive CD34+ AML cells [19] or LSCs [22] after transformation. Concerning interactions with other relevant mutations in AML, FAK expression is lower in patients with FLT3-ITD or RAS mutations suggesting a functional compensation of these pathways [21]. Moreover, other reports have also shown that FAK is constitutively phosphorylated in FLT3ITD- and KITD814V-expressing cells [38].

In contrast to FAK (encoded by the *PTK2* gene), PYK2 (encoded by the *PTK2B* gene) and LYN are predominantly expressed in hematopoietic cells [17,18], including normal CD34+ HPCs and, as described above, they are involved in pro-apoptotic and differentiation processes, so that their expression may identify a subset of patients with a more differentiated phenotype, which shows a better response to therapy. On this basis, the expression of FAK, when PYK2 or LYN expression is absent or low, may confer a highly aggressive phenotype to IR-AML patients, likely also in patients with non-favorable FLT3/NMP1, because their leukemia may contain a higher proportion of progenitor/LSCs, and show higher dissemination and drug resistance capacities. In contrast, PYK2 or LYN overexpression may reflect an AML phenotype with higher differentiation and sensitivity to drugs (in which FAK may not play a relevant role, since its expression lacks prognostic value when PYK or LYN are overexpressed).

We also want to point out some limitations to this study. One of them is the lack of the CEBPA mutation analysis or cohort (almost 50% of patients), which prompted its exclusion from the univariate and multivariate analyses. Nevertheless, CEBPA mutations are rather infrequent [39], so that we believe they are unlikely to have an impact on our analyses. Another limitation is that the prognostic value of the genes studied cannot be extrapolated to patients older than 70 because they have not been included in this study. In addition, since minimal residual disease (MRD) status has been pointed out in recent studies as a strong prognostic factor for relapse and DFS in AML, it would be interesting to analyze the correlation between *PTK2B*, *LYN* and *PTK2* expression and MRD status in the future. Unfortunately, the lack of MRD data for most of the studied patient cohort precluded this analysis. 

## 4. Materials and Methods 

### 4.1. Patients

A total of 324 patients from Hospital de la Santa Creu i Sant Pau (Barcelona, Spain), Hospital La Fe (Valencia, Spain) and Hospital Universitario de Salamanca (Salamanca, Spain), participated in this study and were treated according to the CETLAM-03 (Grupo Cooperativo para el Estudio y Tratamiento de las Leucemias Agudas y Mielodisplasias) or PETHEMA (Programa de Estudio y Tratamiento de las Hemopatías Malignas, trials LMA99, LMA2007 and LMA2010) protocols. In brief, eligible patients were treated with intensive chemotherapy in which induction consisted in a combination of an anthracycline plus cytarabine with or without etoposide, or a combination of idarubicin, cytarabine and etoposide, depending on the protocol. On achievement of complete remission (CR), patients proceeded to consolidation therapy and eligible cases were selected for autologous or allogeneic stem cell transplantation.

Patients included in this study were adults up to the age of 70 with *de novo* IR-AML, according to the Medical Research Council (MRC) classification [40,41]. All samples were collected at diagnosis after obtaining informed written consent in accordance with the Declaration of Helsinki and the Ethics Committee approval of each participating institution on the 21 April 2010 (Ethical Code: 10/030/1069). All patients were selected based on availability of bone marrow specimens. Clinical outcomes between selected and non-selected patients were similar, since they did not present significant differences. Patients older than 70 or with an acute promyelocytic leukemia at diagnosis (M3 in FAB classification) were excluded from the study. The main patient characteristics are shown in Figure 1 and Table S1. 

### 4.2. Time-Dependent Clinical Outcome Endpoints

Overall survival (OS) was calculated as the time (months) from patient diagnosis to death or the last date of follow-up (dead vs. alive) and disease-free survival (DFS) from the complete remission date to relapse/death or last date of follow-up (dead or relapsed vs. alive patients). Finally, cumulative incidence of relapse (CIR) was calculated as the time (months) from the complete remission date to relapse or last date of follow-up or death (competing risk analysis: Relapsed vs. alive non-relapsed vs. dead non-relapsed patients).

### 4.3. RNA Extraction and Gene Expression Analyses

Samples were obtained from bone marrow patient aspirates at diagnosis. Mononuclear cells were isolated by Ficoll-Hypaque gradient and total RNA was extracted using TRIzol reagent (Thermo Fisher Scientific, Waltham, MA, USA) following the manufacturer’s protocol. cDNA was generated after reverse transcription of 1.5 µg total RNA using the High Capacity cDNA Reverse Transcription Kit (Applied Biosystems (AB), Foster City, CA, USA). *PTK2B* (gene that encodes the PYK2 protein), *PTK2* (gene that encodes the FAK protein), *SRC* and *LYN* expression (Hs00169444_m1, Hs00178587_m1, Hs01082246_m1 and Hs00176719_m1, resp. AB) was determined by real-time PCR using the platform ABI 7900HT Fast Real-Time PCR System (AB). Each sample was assessed by triplicate using the TaqMan Gene Expression Assay (AB). The comparative cycle threshold DCt (Ct_target_-Ct_control_) method was used to determine relative expression levels [42]. *ABL* was used as the endogenous control gene (probe: ENP1043, primers: P3035: TGGAGATAACACTCTAAGCATAACTAAAGGT, P3036: GATGTAGTTGCTTGGGACCCA; AB) using VIC fluorophore. Quantitative methods analyses were detailed in previous reports also using *ABL* as control gene [43].

### 4.4. Statistical Analysis

Statistical analyses were performed using the IBM SPSS Statistics program (Release 22.0.0.0, New York, NY, USA). The association between the gene expression and clinical data were analyzed using the Fischer exact test or χ^2^ test. Differences between continuous variables were analyzed with the Student’s t-test or the Mann-Whitney U test in those showing normal or non-normal distribution, respectively. Spearman rank correlation test was used to analyze correlation between variables with non-normal distribution indicating the correlation coefficient (*ρ*) and the *p*-value. To define over or underexpression of *PTK2B*, *LYN*, *PTK2* and *SRC*, a cutoff point was selected using the value of the area under the ROC (Receiver Operating Characteristic) curves. In case of low specificity and sensitivity, exploratory univariate analyses were performed using the mean, the median or the quartiles as thresholds. After these analyses, the third quartile (75th percentile) was selected as cutoff to dichotomize gene expression. Although the mean, the median or other quartiles showed some significant results, the third quartile was the best cutoff obtaining the greater significant differences. The analyzed independent variables included age, gender, white blood cell count (WBC) at diagnosis, karyotype, and molecular alterations (FLT3/ITD and NPM1). Time-dependent endpoints, including OS, DFS and CIR were assessed, and the endpoints to establish the threshold were death or relapse. Time-dependent outcomes were calculated using Kaplan–Meier curves (OS or DFS), and the log-rank test was used for comparisons [44]. The regression model used in univariate and multivariate analyses was the Cox test (OS and DFS). The statistical package R Studio (Version 0.98-1102, 2009–2014, RStudio Inc., Boston, MA, USA) in its R version 3.1.2 (31 October 2014, The R Foundation for Statistical Computing) [45] was used for the competing risk analysis. CIR was analyzed by Gray [46] and Fine and Gray’s tests [47], using R and the *cuminc* and *crr* functions through the *cmprsk* package. After exploratory univariate comparisons, multivariate analyses were performed, including variables with a *p*-value below 0.100 and the INTRO method was used in SPSS to analyze Cox regression. Hazard ratios (HR) with relative 95% confidence interval (CI) are shown in both univariate and multivariate analyses. Differences were considered statistically significant when the *p*-value was < 0.05.

## 5. Conclusions

Our results demonstrate that measuring the expression of the focal adhesion genes *PTK2B*, *LYN* and *PTK2* can refine the intermediate-risk cytogenetic classification of AML, which could improve the stratification of the heterogeneous cytogenetic IR-AML, based on current markers. 

In our cytogenetic IR-AML cohort, *PTK2B* or *LYN* overexpression are independent prognostic factors for OS, DFS and/or CIR. Most importantly, *PTK2B* or *LYN* overexpression has identified a favorable prognosis patient subgroup that would have been associated with a non-favorable outcome, if using only FLT3/NPM1 criteria. In addition, *PTK2* overexpression, concomitantly with *PTK2B* or *LYN* underexpression, identifies a patient subgroup with very poor outcome among IR-AML patients bearing non-favorable FLT3/NPM1 combinations with highly heterogeneous outcome. 

Additional studies in independent IR-AML patient series are necessary to validate *PTK2B* and/or *LYN* overexpression as favorable markers, and *PTK2* as a poor outcome marker. Once validated, these molecular markers may improve patient stratification, as well as the risk-adapted post-remission chemotherapy protocols currently used, especially by ensuring treatment of patients with high probability of response. 

## Figures and Tables

**Table 1:** Univariate and multivariate analyses of the association between relevant clinical variables and focal adhesion gene expression with overall survival (OS) or disease-free survival (DFS) in the cytogenetic IR-AML cohort.

		OS (N = 324)						DFS (N = 282)					
		Univariate			Multivariate			Univariate			Multivariate		
Variable	Item	HR	95% CI	*p*-Value	HR	95% CI	*p*-Value	HR	95% CI	*p*-Value	HR	95% CI	*p*-Value
Age	≤50 years	1						1					
	>50 years	1.947	1.389–2.730	**<0.001**	2.131	1.501–3.025	**<0.001**	1.639	1.158–2.319	**0.005**	1.798	1.254–2.580	**0.001**
Sex	Male	1						1					
	Female	0.953	0.703–1.291	0.756	-	-	-	0.985	0.712–1.362	0.927	-	-	-
WBC	≤20 × 10^9^/L	1						1					
	>20 × 10^9^/L	0.856	0.631–1.161	0.318	-	-	-	0.792	0.571–1.098	0.162	-	-	-
FLT3	FLT3/ITD^−^	1						1					
	FLT3/ITD^+^	1.209	0.871–1.678	0.256	-	-	-	1.231	0.867–1.748	0.246	-	-	-
NPM1	NPM1^−^	1						1					
	NPM1^+^	0.834	0.612–1.138	0.252	-	-	-	0.907	0.652–1.263	0.564	-	-	-
FLT3/NPM1	Favorable	1						1					
	Non-favorable	1.586	1.098–2.292	**0.014**	1.859	1.281–2.699	**0.001**	1.617	1.102–2.372	**0.014**	1.809	1.226–2.668	**0.003**
Karyotype	Normal	1						1					
	Abnormal	1.285	0.905–1.824	0.162	-	-	-	0.972	0.650–1.454	0.890	-	-	-
*PTK2B*	Underexpression	1						1					
	Overexpression	0.589	0.398–0.870	**0.008**	0.630	0.423–0.939	**0.023**	0.552	0.363–0.838	**0.005**	0.585	0.382–0.896	**0.014**
*LYN*	Underexpression	1						1					
	Overexpression	0.621	0.424–0.909	**0.014**	0.649	0.440–0.957	**0.029**	0.569	0.377–0.859	**0.007**	0.596	0.392–0.908	**0.016**
*PTK2*	Underexpression	1						1					
	Overexpression	1.083	0.771–1.520	0.646	-	-	-	1.267	0.886–1.813	0.195	-	-	-
*SRC*	Underexpression	1						1					
	Overexpression	0.981	0.693–1.387	0.913	-	-	-	1.012	0.697–1.468	0.952	-	-	-

Cox test was used to analyze the statistical significance in OS and DFS. *p*-value < 0.05 was considered statistically significant (bold values). “-” indicates that variables were not included in the multivariate analyses (*p*-value > 0.100 in the univariate analysis). HR; hazard ratio. CI; confidence interval.

**Table 2:** Univariate and multivariate analyses of the association between relevant clinical variables and focal adhesion gene expression with cumulative incidence of relapse (CIR) in the cytogenetic IR-AML cohort.

		CIR (N = 282)					
		Univariate			Multivariate		
Variable	Item	HR	95% CI	*p*-Value	HR	95% CI	*p*-Value
Age	≤50 years	1					
	>50 years	1.180	0.779–1.780	0.440	-	-	-
Sex	Male	1					
	Female	1.080	0.725–1.610	0.710	-	-	-
WBC	≤20 × 10^9^/L	1					
	>20 × 10^9^/L	1.070	0.717–1.600	0.740	-	-	-
FLT3	FLT3/ITD^−^	1					
	FLT3/ITD^+^	1.300	0.925–1.820	0.130	-	-	-
NPM1	NPM1^−^	1					
	NPM1^+^	0.819	0.576–1.160	0.270	-	-	-
FLT3/NPM1	Favorable	1					
	Non-favorable	1.920	1.190–3.090	**0.007**	1.935	1.198–3.127	**0.007**
Karyotype	Normal	1					
	Abnormal	0.795	0.500–1.260	0.330	-	-	-
*PTK2B*	Underexpression	1					
	Overexpression	0.426	0.246–0.738	**0.002**	0.449	0.258–0.781	**0.005**
*LYN*	Underexpression	1					
	Overexpression	0.662	0.406–1.080	0.098	0.650	0.391–1.080	0.097
*PTK2*	Underexpression	1					
	Overexpression	0.838	0.525–1.340	0.460	-	-	-
*SRC*	Underexpression	1					
	Overexpression	0.953	0.597–1.520	0.840	-	-	-

Gray and Fine and Gray tests for CIR were used to analyze the statistical significance. *p*-value < 0.05 was considered statistically significant (bold values). “-” indicates that variables were not included in the multivariate analyses (*p*-value > 0.100 in the univariate analysis).

**Table 3:** Association between *PTK2B* or *LYN* expression level and the main clinical and molecular characteristics of the cytogenetic IR-AML patient cohort.

	Cohort	*PTK2B*			*LYN*		
Parameter	Total (N = 324)	Under-Expressed	Over-Expressed	*p*-Value	Under-Expressed	Over-Expressed	*p*-Value
Age, median (range)	55 (17–70)	243	81	0.309 ^‡^	243	81	0.623 ^‡^
≤50 years (%)	121 (37)	86	35	0.233	90	31	0.895
>50 years (%)	203 (63)	157	46		153	50	
Sex, (%)							
Male	172 (53)	129	43	1.000	132	40	0.444
Female	152 (47)	114	38		111	41	
WBC, median (range)	20 (0.03–325)	240	81	0.138 ^‡^	240	81	0.066 ^‡^
≤20 × 10^9^/L (%)	161 (50)	124	37	0.371	128	33	0.055
>20 × 10^9^/L (%)	160 (50)	116	44		112	48	
Unknown	3 (<1)						
Karyotype, (%)							
Normal	247 (76)	190	57	0.091	183	64	0.643
Abnormal	72 (22)	48	24		56	16	
Unknown	5 (2)						
Protein mutations, (%)							
FLT3/ITD^−^	220 (68)	167	53	0.575	170	50	0.161
FLT3/ITD^+^	96 (30)	70	26		67	29	
Unknown	8 (2)						
NPM1^−^	157 (48)	116	41	0.696	122	35	0.242
NPM1^+^	155 (48)	118	37		111	44	
Unknown	12 (4)						
FLT3^−^/NPM1^+^	92 (28)	69	23	0.311 *	66	26	0.177 *
FLT3^−^/NPM1^−^	125 (39)	96	29		101	24	
FLT3^+^/NPM1^−^	31 (10)	19	12		20	11	
FLT3^+^/NPM1^+^	63 (19)	49	14		45	18	
Unknown	13 (4)						
FLT3^−^/NPM1^+^ (Fav.)	92 (28)	69	23	1.000	66	26	0.477
Others (Non-fav.)	219 (68)	164	55		166	53	
Unknown	13 (4)						
Patient status, (%)							
Alive	156 (48)	106	50	**0.007**	108	48	**0.029**
Death	168 (52)	137	31		135	33	
No relapsed	211 (65)	148	63	**0.007**	154	57	0.259
Relapsed	97 (30)	82	15		77	20	
Missing	16 (5)						
Complete Remission	258 (80)	195	63	0.739	191	67	0.620
Resistance or death	59 (18)	43	16		46	13	
Missing	7 (2)						

Results are presented as the number of patients for each characteristic. The percentage of the patients is indicated in brackets for each condition. Fischer exact test was used for most categorical variables (no symbol indicated) to analyze the statistical significance of the differences among studied parameters. For the variables on which Fischer exact test could not be estimated, the χ^2^ test (*) was used. Moreover, for continuous variables showing non normal distribution, the Mann-Whitney U test (^‡^) was used. *p* < 0.05 indicates statistical significance (bold values).

**Table 4:** Univariate and multivariate analyses of the association between relevant clinical variables and focal adhesion gene expression with OS and DFS in patients with non-favorable FLT3/NPM1 combinations with cytogenetic IR-AML.

		OS (N = 219)						DFS (N = 188)					
		Univariate			Multivariate			Univariate			Multivariate		
Variable	Item	HR	95% CI	*p*-Value	HR	95% CI	*p*-Value	HR	95% CI	*p*-Value	HR	95% CI	*p*-Value
Age	≤50 years	1						1					
	>50 years	1.913	1.319–2.774	**0.001**	1.873	1.291–2.716	**0.001**	1.707	1.151–2.533	**0.008**	1.673	1.127–2.483	**0.011**
Sex	Male	1						1					
	Female	0.885	0.618–1.265	0.502	-	-	-	0.921	0.626–1.354	0.675	-	-	-
WBC	≤20 × 10^9^/L	1						1					
	>20 × 10^9^/L	0.868	0.609–1.239	0.436	-	-	-	0.939	0.639–1.380	0.749	-	-	-
FLT3	FLT3/ITD^−^	1						1					
	FLT3/ITD^+^	0.993	0.693–1.422	0.969	-	-	-	1.021	0.693–1.502	0.918	-	-	-
NPM1	NPM1^−^	1						1					
	NPM1^+^	1.180	0.799–1.742	0.406	-	-	-	1.400	0.932–2.103	0.105	-	-	-
Karyotype	Normal	1						1					
	Abnormal	1.065	0.714–1.590	0.757	-	-	-	0.789	0.496–1.256	0.318	-	-	-
*PTK2B*	Underexpression	1						1					
	Overexpression	0.583	0.370–0.918	**0.020**	0.605	0.384–0.953	**0.030**	0.584	0.359–0.951	**0.031**	0.601	0.369–0.979	**0.041**
*LYN*	Underexpression	1						1					
	Overexpression	0.618	0.396–0.966	**0.035**	0.625	0.400–0.977	**0.039**	0.527	0.320–0.866	**0.012**	0.532	0.324–0.875	**0.013**
*PTK2*	Underexpression	1						1					
	Overexpression	1.065	0.715–1.587	0.757	-	-	-	1.168	0.764–1.784	0.473	-	-	-
*SRC*	Underexpression	1											
	Overexpression	0.894	0.592–1.349	0.593	-	-	-	0.900	0.574–1.411	0.646	-	-	-

Cox test was used to analyze the statistical significance in OS and DFS. *p*-value < 0.05 was considered statistically significant (bold values). “-” indicates that variables were not included in the multivariate analyses (*p*-value > 0.100 in the univariate analysis).

**Table 5:** Univariate and multivariate analyses of the association between relevant clinical variables and focal adhesion gene expression with CIR in patients with cytogenetic IR-AML and non-favorable FLT3/NPM1 combinations.

		CIR (N = 188)					
		Univariate			Multivariate		
Variable	Item	HR	95% CI	*p*-Value	HR	95% CI	*p*-Value
Age	≤50 years	1					
	>50 years	1.190	0.746–1.880	0.470	1.170	0.734–1.865	0.510
Sex	Male	1					
	Female	1.200	0.757–1.900	0.440	-	-	-
WBC	≤20 × 10^9^/L	1					
	>20 × 10^9^/L	1.310	0.830–2.080	0.250	-	-	-
FLT3	FLT3/ITD^−^	1					
	FLT3/ITD^+^	1.220	0.766–1.930	0.410	-	-	-
NPM1	NPM1^−^	1					
	NPM1^+^	1.350	0.817–2.240	0.240	-	-	-
Karyotype	Normal	1					
	Abnormal	0.737	0.431–1.260	0.270	-	-	-
*PTK2B*	Underexpression	1					
	Overexpression	0.440	0.238–0.814	**0.009**	0.442	0.238–0.818	**0.009**
*LYN*	Underexpression	1					
	Overexpression	0.569	0.317–1.020	0.059	0.573	0.319–1.030	0.062
*PTK2*	Underexpression	1					
	Overexpression	0.728	0.428–1.240	0.240	-	-	-
*SRC*	Underexpression	1					
	Overexpression	0.838	0.482-1.460	0.530	-	-	-

Gray and Fine and Gray tests for CIR were used to analyze the statistical significance. *p*-value < 0.05 was considered statistically significant (bold values). “-” indicates that variables were not included in the multivariate analyses (*p*-value > 0.100 in the univariate analysis). Age, as a clinical variable, was included in multivariate analysis even with a *p*-value > 0.100 because of its demonstrated strong prognostic value in OS and DFS analyses.

**Table 6:** Univariate and multivariate analyses of the association between relevant clinical variables and PTK2 expression with OS and DFS in patients with cytogenetic IR-AML and non-favorable FLT3/NPM1 combinations that underexpress PTK2B.

		OS (N = 164)						DFS (N = 141)					
		Univariate			Multivariate			Univariate			Multivariate		
Variable	Item	HR	95% CI	*p*-Value	HR	95% CI	*p*-Value	HR	95% CI	*p*-Value	HR	95% CI	*p*-Value
Age	≤50 years	1						1					
	>50 years	1.717	1.140–2.585	**0.010**	1.708	1.135–2.572	**0.010**	1.627	1.048–2.525	**0.030**	1.498	0.957–2.346	0.077
Sex	Male	1						1					
	Female	0.885	0.594–1.319	0.549	-	-	-	0.977	0.635–1.502	0.914	-	-	-
WBC	≤20 × 10^9^/L	1						1					
	>20 × 10^9^/L	1.003	0.676–1.488	0.988	-	-	-	1.083	0.706–1.662	0.714	-	-	-
FLT3	FLT3/ITD^−^	1						1					
	FLT3/ITD^+^	1.185	0.797–1.762	0.401	-	-	-	1.299	0.847–1.994	0.231	-	-	-
NPM1	NPM1^−^	1						1					
	NPM1^+^	1.179	0.770–1.803	0.449	-	-	-	1.498	0.958–2.341	0.076	1.517	0.958–2.401	0.075
Karyotype	Normal	1						1					
	Abnormal	0.928	0.582-1.482	0.756	-	-	-	0.677	0.392–1.169	0.161	-	-	-
*PTK2*	Underexpression	1						1					
	Overexpression	1.548	1.023–2.343	**0.039**	1.538	1.017–2.328	**0.042**	1.686	1.089–2.610	**0.019**	1.758	1.131–2.734	**0.012**

COX test was used to analyze the statistical significance in OS and DFS. *p*-value < 0.05 was considered statistically significant (bold values). “-” indicates that variables were not included in the multivariate analyses (*p*-value > 0.100 in the univariate analysis).

**Table 7:** Univariate and multivariate of the association between relevant clinical variables and *PTK2* expression with OS and DFS in patients with cytogenetic IR-AML and non-favorable FLT3/NPM1 combinations that underexpress *LYN*.

		OS (N = 164)						DFS (N = 141)					
		Univariate			Multivariate			Univariate			Multivariate		
Variable	Item	HR	95% CI	*p*-Value	HR	95% CI	*p*-Value	HR	95% CI	*p*-Value	HR	95% CI	*p*-Value
Age	≤50 years	1						1					
	>50 years	1.928	1.272–2.922	**0.002**	1.868	1.232–2.834	**0.003**	1.722	1.111–2.670	**0.015**	1.556	0.995–2.432	0.052
Sex	Male	1						1					
	Female	0.733	0.487–1.101	0.135	-	-	-	0.796	0.517–1.225	0.299	-	-	-
WBC	≤20 × 10^9^/L	1						1					
	>20 × 10^9^/L	0.901	0.605–1.341	0.607	-	-	-	1.007	0.658–1.541	0.975	-	-	-
FLT3	FLT3/ITD^−^	1						1					
	FLT3/ITD^+^	1.031	0.687–1.546	0.884	-	-	-	1.236	0.806–1.895	0.332	-	-	-
NPM1	NPM1^−^	1						1					
	NPM1^+^	1.199	0.769–1.869	0.423	-	-	-	1.505	0.957–2.367	0.077	1.584	0.991–2.531	0.054
Karyotype	Normal	1						1					
	Abnormal	1.013	0.650–1.581	0.954	-	-	-	0.714	0.428–1.191	0.196	-	-	-
*PTK2*	Underexpression	1						1					
	Overexpression	1.669	1.098–2.536	**0.016**	1.600	1.052–2.433	**0.028**	1.722	1.123–-2.641	**0.013**	1.815	1.172–2.812	**0.008**

Cox test was used to analyze the statistical significance in OS and DFS. *p*-value < 0.05 was considered statistically significant (bold values). “-” indicates that variables were not included in the multivariate analyses (*p*-value > 0.100 in the univariate analysis).

## Supplementary Materials

The following are available online at http://www.mdpi.com/2072-6694/10/11/436/s1, Table S1: Main clinical characteristics of the studied cytogenetic IR-AML patient cohort. Description: Results are presented as the number of patients for each characteristic. The percentage of the patients is indicated in brackets for each condition. WBC; White blood cells. FAB; French-American-British. WHO; World Health Organization. Fav; Favorable. Non-fav; Non-favorable. Table S2: Time-dependent endpoints analyses regarding clinical characteristics and molecular alterations of the cytogenetic IR-AML cohort of our study. Description: The results for the median of follow up are presented as the number of patients (range) for each condition; for the patient status as the number of patients (%); and for all the others as the estimates in percentage ± the standard error. Log-rank test was used to analyze the statistical significance for each variable. *p* < 0.05 was considered statistically significant (bold values). NS represents not significant differences, and the estimates were not included in the table. CR; Complete remission. OS; Overall survival. DFS; Disease-free survival. CIR; Cumulative incidence of relapse. Table S3: Time-dependent endpoints analyses regarding clinical characteristics of the cytogenetic IR-AML with non-favorable FLT3/NPM combination included in our patient cohort. Description: The results for the median of follow up are presented as the number of patients (range) for each condition; for the patient status as the number of patients (%); and for all the others as the estimates in percentage ± the standard error. Log-rank test was used to analyze the statistical significance for each variable. *p* < 0.05 was considered statistically significant (bold values). NS represents not significant differences, and the estimates were not included in the table. CR; Complete remission. OS; Overall survival. DFS; Disease-free survival. CIR; Cumulative incidence of relapse.

## Endnotes

This article was written following the REMARK guidelines [48,49].
