# Supplementary material for: Focal Adhesion Genes Refine the Intermediate-Risk Cytogenetic Classification of Acute Myeloid Leukemia
Source: Cancers (Basel). 2018 Nov 13;10(11):436. doi: 10.3390/cancers10110436 (PMC6265715; doi:10.3390/cancers10110436)
Supplement: Supplementary file 1 [file cancers-10-00436-s001.pdf]

Supplementary Materials: Focal Adhesion Genes Refine the Intermediate-Risk Cytogenetic Classification of Acute Myeloid Leukemia

Victor Pallarès, Montserrat Hoyos, M. Carmen Chillón, Eva Barragán, M. Isabel Prieto Conde, Marta Llop, Aïda Falgàs, María Virtudes Céspedes, Pau Montesinos, Josep F. Nomdedeu, Salut Brunet, Miguel Ángel Sanz, Marcos González-Díaz, Jorge Sierra, Ramon Mangues and Isolda Casanova

Table S1. Main clinical characteristics of the studied cytogenetic IR-AML patient cohort.

Parameter	Cohort Total (N = 324)	Parameter	Cohort Total (N = 324)
Age, median (range)	55 (17–70)	Protein mutations, (%)	
≤50 years (%)	121 (37)	FLT3/ITD$^-$	220 (68)
>50 years (%)	203 (63)	FLT3/ITD$^+$	96 (30)
Sex, (%)			
Male	172 (53)	NPM1$^-$	157 (48)
Female	152 (47)	NPM1$^+$	155 (48)
WBC, median (range)	20 (0.03–325)		
≤20 × 10^9/L (%)	161 (50)	FLT3$^-$/NPM1$^+$	92 (28)
>20 × 10^9/L (%)	160 (50)	FLT3$^-$/NPM1$^-$	125 (39)
Unknown	3 (<1)	FLT3$^+$/NPM1$^-$	31 (10)
FAB Classification, (%)		Unknown	13 (4)
M0	27 (8)		
M1	80 (25)	FLT3$^-$ + NPM1$^+$ (Fav.)	92 (28)
M2	58 (18)	Others (Non-fav.)	219 (68)
M4	59 (18)	Unknown	13 (4)
M4E	2 (<1)		
M5	74 (23)	CEBPA$^-$	156 (49)
M6	8 (2)	CEBPA$^+$	15 (5)
M7	1 (<1)	Unknown	148 (46)
Unknown	15 (5)		
WHO Classification, (%)		MLL Rearranged	5 (1)
AML with recurrent genetic abnormalities	121 (37)	Unknown	226 (70)
AML with myelodysplasia-related changes	10 (3)		
AML with minimal differentiation	19 (6)		
AML without maturation	45 (14)	Patient status, (%)	
AML with maturation	28 (9)	Alive	156 (48)
Acute myelomonocytic leukemia	32 (10)	Death	168 (52)
Acute monoblastic/monocytic leukemia	43 (13)		
Acute erythroid leukemia	3 (1)	Recurrences, (%)	
Acute megakaryoblastic leukemia	1 (0)	Non-relapsed	211 (65)
Therapy-related myeloid neoplasms	4 (1)	Relapsed	97 (30)
Non-classifiable AML	11(3)	Missing	16 (5)
Missing	7 (2)		
Karyotype, (%)		Complete Remission	258 (80)
Normal	247 (76)	Resistance or death	59 (18)
Abnormal	72 (22)	Missing	7 (2)
Unknown	5 (2)		

Results are presented as the number of patients for each characteristic. The percentage of the patients is indicated in brackets for each condition. WBC; White blood cells. FAB; French-American-British. WHO; World Health Organization. Fav; Favorable. Non-fav; Non-favorable.

Table S2. Time-dependent endpoints analyses regarding clinical characteristics and molecular alterations of the cytogenetic IR-AML cohort of our study.

Parameter	OS	DFS	CIR
	**N = 324**	**N = 282**	**N = 282**
Median follow-up in months			
Alive, (Range)	55 (1.7–154)		
Alive and CR patients, (Range)		47 (0.3–153)	47 (0.3–153)
Survival or CIR at 5 years, percent	45.4 ± 2.9	44.9 ± 3.2	36.7 ± 3.1
Patient status, patient number			
Alive (%)	156 (48)	153 (54)	153 (54)
Death (%)	168 (52)	129 (46)	129 (46)
Relapsed (%)	97 (30)	96 (34)	96 (34)
Age, percent	**$p < 0.001$**	**$p = 0.005$**	
≤50 years	58.3±4.7	54.3±5.0	NS
>50 years	32.9±4.5	32.2±4.9	NS
FLT3/NPM1 Combination, percent	**$p = 0.013$**	**$p = 0.013$**	**$p = 0.008$**
$FLT3^{-}$ + $NPM1^{+}$ (Favorable)	53.6 ± 7.1	48.7 ± 7.0	32.6 ± 6.9
Others (Non-favorable)	39.0 ± 3.7	38.5 ± 4.2	42.1 ± 4.0

Patient follow up is presented as median and range in months for each condition; patient status as the number and percent of patients; and for the other parameters as the estimates in percentage ± the standard error. Log-rank test was used to analyze the statistical significance for each variable. $p < 0.05$ was considered statistically significant (Bold values). NS represents not significant differences, and the estimates were not included in the table. CR; Complete remission. OS; Overall survival. DFS; Disease-free survival. CIR; Cumulative incidence of relapse.

Table S3. Time-dependent endpoints analyses regarding clinical characteristics of the cytogenetic IR-AML with non-favorable FLT3/NPM combination included in our patient cohort.

Parameter	OS	DFS	CIR
	**N = 219**	**N = 188**	**N = 188**
Median follow-up in months			
Alive, (Range)	55 (1.7–154)		
Alive and CR patients, (Range)		50 (0.3–153)	50 (0.3–153)
Survival or CIR at 5 years, percent	40.4 ± 3.5	41.9 ± 3.8	40.4 ± 3.8
Patient status, patient number			
Alive (%)	96 (44)	93 (50)	93 (50)
Death (%)	123 (56)	95 (50)	95 (50)
Relapsed (%)	73 (33)	72 (40)	72 (40)
Age, percent	**$p < 0.001$**	**$p = 0.007$**	
≤50 years	51.3 ± 5.4	50.7 ± 5.6	NS
>50 years	29.0 ± 5.0	27.4 ± 5.9	NS

Patient follow up is presented as median and range in months for each condition; patient status as the number and percent of patients; and for the other parameters as the estimates in percentage ± the standard error. Log-rank test was used to analyze the statistical significance for each variable. $p < 0.05$ was considered statistically significant (Bold values). NS represents not significant differences, and the estimates were not included in the table. CR; Complete remission. OS; Overall survival. DFS; Disease-free survival. CIR; Cumulative incidence of relapse.

© 2018 by the authors. Licensee MDPI, Basel, Switzerland. This article is an open access article distributed under the terms and conditions of the Creative Commons Attribution (CC BY) license (http://creativecommons.org/licenses/by/4.0/).
